# Pain in elderly people with severe dementia: A systematic review of behavioural pain assessment tools

**DOI:** 10.1186/1471-2318-6-3

**Published:** 2006-01-27

**Authors:** Sandra MG Zwakhalen, Jan PH Hamers, Huda Huijer Abu-Saad, Martijn PF Berger

**Affiliations:** 1 Department of Health Care Studies, Section of Nursing Science, Universiteit Maastricht, P.O. Box 616, 6200 MD Maastricht, The Netherlands; 2 School of nursing, Faculty of Medicine, American University of Beirut, P.O.Box 11-0236, Riad El-Solh / Beirut 1107 2020, Lebanon; 3 Department of Methodology and Statistics, Universiteit Maastricht, The Netherlands

## Abstract

**Background:**

Pain is a common and major problem among nursing home residents. The prevalence of pain in elderly nursing home people is 40–80%, showing that they are at great risk of experiencing pain. Since assessment of pain is an important step towards the treatment of pain, there is a need for manageable, valid and reliable tools to assess pain in elderly people with dementia.

**Methods:**

This systematic review identifies pain assessment scales for elderly people with severe dementia and evaluates the psychometric properties and clinical utility of these instruments. Relevant publications in English, German, French or Dutch, from 1988 to 2005, were identified by means of an extensive search strategy in Medline, Psychinfo and CINAHL, supplemented by screening citations and references. Quality judgement criteria were formulated and used to evaluate the psychometric aspects of the scales.

**Results:**

Twenty-nine publications reporting on behavioural pain assessment instruments were selected for this review. Twelve observational pain assessment scales (DOLOPLUS2; ECPA; ECS; Observational Pain Behavior Tool; CNPI; PACSLAC; PAINAD; PADE; RaPID; Abbey Pain Scale; NOPPAIN; Pain assessment scale for use with cognitively impaired adults) were identified. Findings indicate that most observational scales are under development and show moderate psychometric qualities.

**Conclusion:**

Based on the psychometric qualities and criteria regarding sensitivity and clinical utility, we conclude that PACSLAC and DOLOPLUS2 are the most appropriate scales currently available. Further research should focus on improving these scales by further testing their validity, reliability and clinical utility.

## Background

In the last decade, there has been a growing interest in pain among elderly people. Pain among nursing home residents is a common and major problem. Statistics indicate that pain is twice as likely to occur in individuals aged 60 and older as in younger persons [1-3]. The prevalence of pain in elderly nursing home residents is 40–80% [4-9], showing that they are at great risk of experiencing pain. As in most countries, the population of individuals over the age of 65 in the Netherlands is growing fast. Demographic trends make it likely that in 2040, 22.9% of the population will belong to this category [10].

More than 50% of US nursing home residents have substantial cognitive impairment or dementia [6]. This situation is comparable to that in European countries like Austria and the UK, where dementia has been found to occur in over 60% of the institutionalised population [11,12] and in the Netherlands, where more than half of the nursing home residents have been diagnosed with dementia [13]. This demographic data suggests that the volume of nursing home care required will increase and pain assessment will be a major challenge.

Dementia, caused by a variety of conditions, has been defined as a complex of symptoms characterized by progressive global deterioration of cognitive functioning [14]. The impairment level is often categorized by means of Mini Mental State Examination (MMSE) scores. The MMSE assesses orientation, registration, attention and recall, and language [15]. Dementia causes serious and unique barriers to pain assessment and can be characterized by memory loss, personality changes and loss of other functions such as judgement, abstract thinking and language skills. Furthermore, common behaviours associated with pain may be absent or difficult to interpret [16]. On the other hand, symptoms attributed to dementia may actually be an indication of pain. For example, aggressive behaviours may be a protective response by subjects who are not able to articulate their pain [17]. Such behaviour, however, could also be mistaken for a symptom of dementia. As a result of these problems, which affect almost all dementia patients, pain in this population is extremely difficult to assess.

There is evidence that pain assessment is currently inadequate and that elderly people with dementia are being undertreated [5,6,18-21]. This undertreatment could lead to various additional problems like cognitive (e.g. concentration problems) and behavioural symptoms (e.g. aggression or depression) at patient level, as well as to greater and heavier demands on caregivers and increased care demands and costs at organization level. The main reason for undertreatment is underdetection [4,5]. Existing evidence of underreported, underdetected and undertreated pain among people with dementia provides the clearest argument for the urgent need to use a pain assessment scale regularly. Assessment to detect pain is thus essential, and is the first crucial step towards adequate treatment of geriatric pain patients with dementia [22]. There is therefore a need for manageable, valid and reliable pain assessment tools.

Pain can be assessed by means of self-reports, behavioural or physiological measures. Self-reporting is often considered as the 'gold standard' in pain assessment. A broad range of self-report scales is currently available to assess pain in the elderly, most of which have been developed for and tested in a different setting before being used among elderly people with dementia [23]. The most frequently assessed component of pain is pain intensity. Commonly used measures of pain intensity include Visual Analogue Scales (VAS), Verbal Rating Scales (VRS), Numeric Rating Scales (NRS) and Facial Pain Scales (FPS) [23,24]. It is generally worth noting that elderly people find it more difficult to use self-report scales correctly than younger adults [22], and no single self-report scale seems appropriate for all elderly people. Self-report scales require the capacity to understand the task and to communicate about the pain experienced. Increasing age has been associated with difficulties in abstract thinking, which makes it more difficult to use scales requiring this cognitive skill. This also implies that self-reported scores might be affected and influenced by context and are not always that solid.

A substantial proportion of elderly people living in institutions are unable to understand and answer even simple yes/no questions, and therefore cannot self-report pain [25,6]. In the advanced stages of dementia, when the elderly persons are severely impaired, other methods of assessment, like behavioural pain assessment methods, become more useful and necessary. Developing a tool that can be used to assess their pain may greatly improve the quality of life of the estimated 20–30% of nursing home residents who cannot adequately communicate their needs [26]. Non-verbal assessment tools based on behavioural observation methods include observation of changes in behaviour and functioning, involving sleep, appetite, physical activity, mobility and facial/body language. Physiological measures, like heart rate or blood pressure, can also provide important information, especially in the assessment of non-verbal demented elderly people. Therefore, behavioural pain assessment scales often use physiological indicators in combination with other (e.g. social) indicators. Research into physiological measures has been scarce, due to their limited validity and practical limitations [27]. Physiological responses are often not specific enough to serve as pain indicators, and autonomous physiological responses to pain are difficult to discriminate from other states of distress [28].

In recent years, research in the field of pain among elderly people with severe dementia has increasingly focused on understanding expressions indicating possible pain [4,29,9]. However, there have been a limited number of studies focusing on the development of scales to measure pain in these people, and to our best knowledge no overview was available of behavioural pain assessment scales developed especially to assess pain in elderly people with dementia. The main research questions in the present study were therefore: (1) which behavioural pain assessment tools are available to assess pain in elderly people with dementia and (2) what are the psychometric qualities of these tools?

## Methods

We reviewed the relevant publications based on an extensive search strategy, involving computer searches of Medline, Psychinfo and Cumulative Index to Nursing and allied Health Literature (CINAHL) to identify the literature. The keywords we used and the search results are listed in Table 1.

**Table 1:** Search strategy.

Keywords used: (Pain) AND (Scale OR assessment OR measure) AND (Elderly OR residents ORgeriatric OR nursing homes OR cognitive impairment OR dementia OR Alzheimer)			
**Source**	**Hits (N =)**	**Selection based on reading abstracts (N =)**	**Final selection based on publications (N =)**

Databases	Psychinfo 191	29	31
	Medline 977	70*	
	CINAHL*** 219	17**	
Abstracts	8	5	5
Citation/reference screening	37	17	15
Unpublished manuscripts	6	3	3
			
Total		141	54

* *N = 19/70 overlapping with the Psychinfo search*

** *N = 15/17 overlapping with the Psychinfo and Medline searches*

*** *Because of the large number of publications found using CINAHL, our search of this database was limited by using the keyword 'nursing'*

In addition, citations and references in selected journal articles were screened to supplement the search strategy. Unpublished manuscripts were collected by approaching colleagues working in the field of pain among the elderly. Finally, the abstract books of the 7^th^, 8^th^, 9^th ^and 10^th ^International Association for the Study of Pain World Congresses were screened for relevant publications.

The review focused on publications that have appeared over the last 17 years, from 1988 to January 2005. The following selection criteria were used to screen relevant publications:

~ Publications had to describe an assessment instrument/scale for elderly patients with dementia or a subgroup of elderly patients with dementia, for example, Alzheimer patients.

~ The assessment scale had to have been used to measure pain by means of self-reports by patients or behavioural measures.

~ Publications had to be in English, Dutch, German or French.

~ Publications had to be other than case reports or secondary sources/reviews.

Our search yielded a large number of publications (see Table 1). After the abstracts of all publications had been screened, 141 publications remained. Not all of these studies were included in the present review, however, because several were reviews/secondary sources or reported on physiological measures. Our database search finally identified 31 publications, while screening citations and references identified another 15 publications. Five relevant abstracts were found in abstract books and three unpublished manuscripts were included. Eventually, 54 publications were identified as suitable for the review on behavioural assessment and self-report tools. Twenty-nine of these 54 publications referred to 14 behavioural assessment scales, while 29 reported on the use of self-reports. The evaluation of self-report tools will be presented in another article [Zwakhalen et al., in preparation]. Two of the 14 scales were not included in this review. The Discomfort Scale for Patients with Dementia of the Alzheimer Type (DS-DAT) by Hurley et al. [30] was not included because the concept of discomfort measured by the DS-DAT differs from the concept of pain. The Pain Assessment Tool in Confused Older Adults (PATCOA) by Decker & Perry [31] was not included because on closer examination, this tool was found to have been designed for use in a confused but cognitively intact sample of elderly people.

Data abstraction criteria used to evaluate behavioural assessment scales (see Table 2) were partly based on Streiner & Norman's requirements for health measurement scales [32]. The following data were extracted (if available) to examine the nature and methodological quality of the assessment scales: type of assessment scale (including items of the scale), source of the items (origin), scoring/scaling response, sample size of patients, content validity, information about feasibility (including completion rate of self-report scales and instructions accompanying behavioural scales), homogeneity, reliability and validity. As a quality check, a small part of the data abstraction process (N = 3 articles on behavioural pain assessment scales) was conducted by two reviewers (with overall agreement found to be 90%).

**Table 2:** Criteria used in overall quality judgement

**Aspect**	**Score**
Origin of items	2 if items were specially collected for use in elderly people with dementia/CI
	1 if items were modified for use in elderly people with dementia/CI
	0 if items originated from a scale developed for another population
Number of participants*	2 if N => 100 and the number of elderly people with dementia included was considerable relative to the number of items/variables or 50 < N < 100 and corrected for multiple testing
	1 if 50 < N < 100 and the number of elderly people with dementia included was considerable relative to the number of items/variables or N < 50 and corrected for multiple testing
	0 if N < 50, not corrected for multiple testing and small number of elderly people with dementia included
Content validity	2 if scale seems to cover all important items/dimensions (in the reviewers' opinion): pain items were collected for the specific population and different sources/methods were used to collect items
	1 if the scale seems to cover important items/dimensions to a moderate extent (in the reviewers' opinion): items were adapted to the population and different sources/methods were used to collect items
	0 if the scale does not seem to cover the important items/dimensions (in the reviewers' opinion)
Criterion validity	2 if correlates acceptable to high (r > .60) according to the 'gold standard' or acceptable according to a 'silver standard' and sensitivity/specificity is determined to be acceptable
	1 if correlates moderate-acceptable (.40 < r < .60) according to the 'gold standard' or acceptable according to a 'silver standard'
	0 if correlates low (r < .40) or no information is provided
Construct validity in relation to other pain tool	2 if correlates with other pain measures acceptable to high (r > .60) 1 if correlates with other pain measures are moderate (r > .40 < .60) 0 if correlations are low (r <,40) or no information is provided
	
	
Construct validity II differentiation	2 if the scale differentiates well (in the reviewers' opinion) between pain and no pain, calm and distressed, pre- and post-medication etc.
	1 if the scale differentiates moderately well (in the reviewers' opinion) between pain and no pain, pre- and post-medication
	0 if the scale does not differentiate or no information is provided
Homogeneity*	2 if .70 < alpha < .90
	1 if alpha > .90 or > .60 alpha <.70
	0 if alpha < .60 or no information is provided
Inter-rater reliability # $	2 if reliability coefficient >.80
	1 if .60 < reliability coefficient < .80
	0 if reliability coefficient < .60 or no information is provided
Intra-rater and/or test-retest reliability $	2 if reliability coefficient >.80 1 if .60 < reliability coefficient < .80
	
	0 if reliability coefficient < .60 or no information is provided
Feasibility	2 if scale is short, manageable with instructions, scoring interpretation
	1 if scale is manageable (one format)
	0 if scale is more complex

Total score ranges from 0 to 20

# The type of reliability analysis is not specified in the criteria used, although it influences the value of the coefficient

* The number of items in the scale is not specified in relation to this criterion although it influences the value of the coefficient

$ item scores 0 if based on interview (instead of e.g. behavioural observations)

## Results

An extensive literature search traced 12 behavioural pain scales, each of which is described below. Specifically, information is provided about the name of the scale, its origin, the number of items/dimensions, the setting, the scoring method/range and practical aspects. In addition, Table 3 presents information on psychometric qualities of the behavioural scales (partly based on Streiner and Norman's criteria for health measurement scales [32]). The assessment scales are discussed in chronological order.

**Table 3:** Psychometric qualities of behavioural pain assessment scales for elderly people with a cognitive impairment

**Assessment tool/source**	**Dimensions/items Scoring range**	**Origin of items**	**Number of participants/CI participants**	**Validity**				**Homogeneity IC**	**Reliability**		**Feasibility**	**Overall judgement **(range 0–20)
								
				**Content**	**Criterion**	**Construct I **relation other pain tools	**Construct II **differentiates		**Inter-rater**	**Intra-rater or Test-retest**		
**DOLOPLUS 2 **Wary *et al*., (1992 first version, France)	10 items, 3 dimensions somatic (N = 5 items) psychomotor (N = 2) psycho-social (N = 3) scoring range 0–30	modified pain scale for children (DEGR)	N = 510 N> 100 CI (few non-communicative)	y	?	Y VAS-DOLOPLUS r very significant	y ? differs in time	y alpha .82	y kappa = ??	y test-retest	incl. instructions, lexicon scoring interpretation English version available	
		
		**1**	**2**	**1**	**0**	**1**	**1**	**2**	**1**	**1**	**1**	**11**
		
**L'Echelle Comportementale pour Personne Agées (ECPA) **Alix *et al *. (1993, France)	11 items, 3 dimensions pre-care post-care during activities scoring range 0–44	modified pain scale for children (DEGR)	N = 118 N = ?? CI	y	n	y VAS-EPCA Pearson r = .67 (N = 16)	? differs in time factor analysis	y alpha .70	y Intra Class r = .80	?	manageable scale scoring interpretation not available German version available	
		
		**1**	**1**	**1**	**0**	**2**	**1**	**2**	**2**	**0**	**1**	**11**
		
**L'échelle Comportementale simplifiée (l'ECS) **Baulon *et al*. (1995, France)	10 items scoring range 0–14	newly developed, (multidisc. opinion) not specific for CI	N = 146 N = ?? CI	?	n	n	?	?	?	?	incl. instructions, lexicon	
		
		**1**	**1**	**1**	**0**	**0**	**0**	**0**	**0**	**0**	**1**	**4**
		
**The Observational Behavior Tool **Simons & Malabar (1995, UK)	25 items, 7 dimensions verbal response (N = 8) facial expression (N = 3) body language (N = 5) conscious state (N = 3) physiological change (N = 3) behavioural change (N = 1) feedback from others (N = 2) scoring range 0–25	items derived from chronic back pain tool	N = 105 39 of 105 non-verbal	y	n	n	n	n	n	n	? manageable scale scoring interpretation not available	
		
		**0**	**2**	**1**	**0**	**0**	**0**	**0**	**0**	**0**	**1**	**4**
		
**Checklist of Non-Verbal Pain Indicators (CNPI) **Feldt *et al*. (2000, USA)	6 clustered items (rest vs. movement) scoring range 0–6	modified pain scale	N = 88 elderly hip fracture patients 53 CI, 35 NI	y	n	y r VDS/CNPI r = .372 at rest r = .428 movement	y CNPI rest vs movement pre- vs post-operative	y Low, alpha .54–.64	y 93% dichotomous Kappa .63–.82 (N = 12)	n	y short, incl. instructions, scoring interpretation	
		
		**1**	**1**	**1**	**0**	**0**	**1**	**0**	**1**	**0**	**2**	**7**
		
**Pain Assessment Checklist for Seniors with Limited Ability to Communicate (PACSLAC) **Hadjistavropoulos *et al*. (2002, Canada)	60 items, 4 dimensions facial expressions (N = 13) activity/body movements (N = 20) social/personality/mood (N = 12) physiological/eating/sleeping/vocal (N = 15) scoring range 0–60	newly developed for this group of elderly	Study 1 N = 28 nurses Study 2 N = 40 nurses Study 3 N = 40 nurses	y	n	y 0–10 scale/PACSLAC r = .39–.54	y differentiates pain and calm event r = .8 between pain scenes	y moderate-good . 82–.87 total scale . 55–.73 subscales in study 3	n	n (only for transcript interviews .94)	y long but simple list	
		
		**2**	**2**	**2**	**0**	**1**	**1**	**2**	**0**	**0**	**1**	**11**
		
**Pain Assessment in Advanced Dementia (PAINAD) **Warden, Hurley and Volicer. (2002, USA)	5 (categorical) items breathing negative vocalization facial expression body language consolability scoring range 0–10	modified pain scale	N = 19 observed CI N = 25 records	y	n	y VAS/PAINAID Pearson r = .75– .76 DS-DAT/PAINAID Pearson r = .76	y factor analysis differentiates between pleasant and aversive pre/post-medication	y moderate < .70	y Pearson r = .82–.97	n	y categorical but short, manageable scale item explanation incl.	
		
		**1**	**0**	**1**	**0**	**2**	**2**	**1**	**2**	**0**	**2**	**11**
		
**Pain Assessment in Dementing Elderly (PADE) **Villanueva *et al*. (2003, USA)	24 items, 3 parts physically observable facial expressions global pain assessment functional activities	newly developed for this group of elderly (literature, interviews, observations)	Study 1 N = 25 CI Study 2 N = 40 CI	y	n	?	y differentiates between pain and no pain CMAI (agitation)/PADE r = .3–.4	y alpha= 0.24–0.88 part 1 good part 3 low	y Intra Class r study 1 .81–.96 study 2 .54–.96	y test-retest Intra Class r study 1 .34–.89 study 2 .70–.98 (part 2 lowest)	? difficult format due to different scaling (Likert, VAS, Multiple choice), long list	
		
		**2**	**1**	**2**	**0**	**0**	**1**	**1**	**2**	**1**	**0**	**10**
		
**Rating Pain in Dementia (RaPID) **Sign & Orrell. (2003, UK)	18 (clustered) items, 4 dimensions behavioural (N = 11) emotional (N = 2) autonomic (N = 2) postural (N = 3) scoring range 0–54	newly developed for this group of elderly (literature, experts)	N = 48 demented	y	n	y RaPID/McGill/VAS scores r = .8–.86	n	y .79 total scale	y mean .97 based on interviews with caregiver- pat.	y test-retest >.75 for all items based on interviews with caregiver- pat.	y clustered but list of acceptable length scoring interpretation not available	
		
		**1**	**1**	**2**	**0**	**2**	**0**	**2**	**0**	**0**	**1**	**9**
		
**The Abbey Pain Scale **Abbey *et al*. (2004, Australia)	6 (categorical) items vocalisation facial expression change in body language behavioural change physiological change physical change scoring range 0–18	modified pain scale (items derived from Hurley (1992) and Simons & Malabar (1995) Modified by experts trough a Delphi study	Stage 1 N = 52 CI (770 pain episodes) Stage 2 N = 61 CI (236 pain episodes)	y	n	y nurses holistic assessment/Abbey scale r = .59	y differentiates between pre and post intervention	y .74–.81 total scale	y low-modest coefficient = ??	n	y categorical but short, manageable scale	
		
		**1**	**2**	**1**	**0**	**1**	**1**	**2**	**0**	**0**	**2**	**10**
		
**The Non-Communicative Patient's Pain Assessment Instrument (NOPPAIN) **Snow *et al*. (2004, USA)	4 sections/parts e.g. observed daily activities pain response (6 items: words, pain faces, noises, bracing, rubbing, restlessness on a 6 point Likert scale) pain location pain thermometer	(multidisc. expert opinion) No specific information about origin of the items	N = 37 CI in a initial feasibility study N = 21 NA (6 video's)	y	n	y video gold standard/NA ratings kappa = .87 low intensity pain condition had smallest parameters	n	n	n	n	y brief, not time consuming scoring interpretation not available	
		
		**1**	**0**	**1**	**0**	**2**	**0**	**0**	**0**	**0**	**1**	**5**
		
**Pain Assessment Tool for Use with Cognitive Impaired Adults **Davies *et al*. (2004, Australia)	11 sections/parts e.g. existing painful conditions physiological measures self-report of pain facial expression usual behaviours changes in behaviours (5 headings: vocalisation, body posture, activities of daily living, cognitive functioning, physical changes) usual and new comfort measures	newly developed for this group of elderly (literature, experts, focus group discussion)	N = 27 CI N = 14 nurses	y	n	n	n	n	n	n	y difficult format due to different scaling (e.g. body map, physiological and behavioural items), long list, time consuming	
		
		**2**	**0**	**2**	**0**	**0**	**0**	**0**	**0**	**0**	**0**	**4**

? = no clear information/data available CI = cognitively impaired, NI= non-impaired, NA = nursing assistants, y = information provided, n = no information provided

Overall quality judgement by the reviewers (see table 1 for criteria)

The *DOLOPLUS2 *by Wary *et al*. (1992) is a behavioural scale evaluating pain in elderly people [33-35]. The DOLOPLUS2 is available in a French and an English version. Its is unclear whether the English version has been psychometrically tested. The scale is based on the Douleur Enfant Gustave Roussy (DEGR) scale [36] for young children and has been adapted for use in the elderly. It involves observations of patient behaviour in ten different situations (10 items/3 dimensions) that could potentially involve pain. Items include sleep, verbal reaction and problems of behaviour. Each of the ten items can be described at one of four different levels – rated from zero to three – representing increasing intensity of pain [34]. A score greater than or equal to five out of 30 (maximum pain score) confirms pain. The DOLOPLUS2 score does not represent pain experience at a specific moment but reflects on the progression of experienced pain.

Several studies have been conducted in geriatric centres and palliative care units to validate the scale, investigating test-retest reliability, concurrent validity and inter-rater reliability [37,38]. The proportion of non-verbal individuals tested was rather small (1–5% of the sample). According to the authors, the convergent validity of the DOLOPLUS2 and the VAS-*patient *was significant (p < 0.001) and DOLOPLUS2 demonstrated good sensitivity. There was satisfactory stability on the retest. A t-test analysing the intra-observer differences found no significant differences for the total score or for item scores. An inter-rater correlation test between two physicians showed no significant difference (p < 0.001), and good levels of internal consistency (α = 0.82) were found. Closer examination of the scale reveals that little information is provided about several aspects of the tool and tool construction. These limitations include a lack of information on correlation coefficients (inter-rater reliability, test-retest reliability) and of information about the determination of cut-off scores and the impairment level of the participants. In acute settings, its value might be limited because patients must be well known to the nurses who have to complete the DOLOPLUS2, whereas the value of a scale becomes greater if it can be used without in-depth knowledge of the patient [39]. Although the scale is accompanied by a lexicon and instructions for use, and is, according to its authors, easy to use, it is conceivable that nursing home staff may have difficulties interpreting items of the DOLOPLUS2, as certain items seem difficult to understand or interpret. In addition, the scale's clinical utility should also be further tested directly at the bedside in larger samples of non-verbal cognitively impaired elderly patients. A final comment concerns the total pain score and its sensitivity. The DOLOPLUS2 score does not represent pain experienced at a particular moment but reflects the progression of experienced pain. The maximum score on the DOLOPLUS2 is 30, and a score of 5 already represents pain. This raises questions about the scale's specificity.

*L' échelle Comportementale pour Personnes Agées (ECPA) *by Alix *et al*. is a behavioural scale for non-communicating elderly people [40,41]. French and German [42] versions of the ECPA are available, although it is unclear whether the German version has been validated.

This scale was also inspired by the Douleur Enfant Gustave Roussy (DEGR) scale [36] for young children and was adapted for use in the elderly. The scale consists of 11 items with five response modalities scored from 0 to 4, representing increasing degrees of pain. The total score ranges from 0 (no pain) to 44 (absolute pain). Factor analysis showed that the ECPA has three dimensions, defined on the basis of principal component factor analysis: pre-care, post-care and during activities [41]. An example of response modalities comprising the item 'facial expression' is 0 = relaxed face; 1 = concerned face; 2 = face sometimes grimacing; 3 = frightened, face contorted with pain.

The homogeneity of the items (Cronbach's α = .70), convergent validity between the VAS and ECPA (Pearson r = .67, N = 16) and inter-rater reliability (Intra-class reliability = .80) have been preliminarily tested in a sample of hospitalised patients in a long-term stay department [41]. Its clinical value needs to be further examined.

The *Simplified Behavioural Scale (ECS) *was published in 1995 by Baulon and colleagues to detect changes in behaviour in geriatric patients with and without communicative limitations [43]. ECS was designed by a multidisciplinary team of nurses and medical staff. The scale consists of ten items scored on three, four or five levels, depending on the item. The first six items are assessed after care, while items 7 and 8 are assessed during care, and items 9 and 10 every 24 hours. Examples of items included in the scale are sleep, verbal reaction and interaction with the environment. A lexicon and users' instructions for the ECS are available. The scale has not been tested for validity and reliability.

The *Observational Pain Behaviour Tool *by Simons & Malabar (1995) is an assessment tool designed specifically for everyday use with elderly patients in hospital settings [39]. The tool is based on the pain tool described by Keefe and Block [44]. The tool consists of a data sheet, a pain assessment chart and a menu of observable pain behaviours (N = 25) that are to be recorded. These behaviours had been found to discriminate between manifestations of pain and depression in tests using alert adults with chronic low back pain. Scoring is based on entering the behaviour on the sheet as being present at a certain moment and does not include information on pain intensity. Examples of the 25 items included in the scale are 'verbal expression' (e.g. 'ouch'), 'not relaxed, drawn-up knees', and 'drowsy'. The tool has been pilot-tested in 105 elderly hospitalised patients by observing pain behaviours, carrying out pain interventions and re-observing later to verify the effectiveness of the intervention [39]. The authors claim that the tool is practical. The fact that carers without in-depth knowledge of the patient were able to use the tool is an important clinical advantage. Based on the result of the evaluation, further investigation of the tool's validity and reliability is necessary.

*The Checklist of Nonverbal Pain Indicators (CNPI) *by Feldt is a behavioural observation scale for non-verbal residents with severe cognitive impairment [45]. The scale is a modification of the University of Alabama Birmingham Pain Behaviour Scale (UAB PBS), which was designed to measure chronic pain [46], from which some items were eliminated and others redefined. Scoring involves patient observation at rest and during movement. Examples of the six more or less clustered items are 'restlessness', 'rubbing' and 'vocal complaints' (verbal). An item is scored as '1' if the behaviour was observed during activity or rest and as '0' if the behaviour was not observed (range of total scale 0–6). After adding up the two scores (for movement and rest) the interpretation is as follows: '1–2' mild pain, '3–4' moderate pain, '5–6' severe pain [45]. The tool was tested in a convenience sample of hospitalised patients aged 65 and older with a hip fracture. The cognitively impaired group (N = 53) had MMSE scores below 23 (mean = 12.2). The authors claim good face validity. CNPI and the patients Verbal Descriptive Scale correlate significantly, although in the impaired subgroup, CNPI only correlated significantly with the VDS during movements. A more important finding was that these correlations were low (r = .372 at rest; r = .428 during movements). Moderate levels of internal consistency (α = .54 at rest, α = .64 during movement) and good inter-rater reliability (IR agreement 93%) were found on the dichotomous checklist (although measured in a relatively small sample, N = 13). Based on reported findings, the CNPI has poor psychometric qualities. Therefore, further development of the scale and psychometric testing (e.g. inter-rater reliability, test-retest reliability) in larger populations seems essential.

The *Pain Assessment Checklist for Seniors with Limited Ability to Communicate (PACSLAC) *by Fuchs et al. intends to be a clinically useful scale for assessing pain in patients with dementia [47,48]. PACSLAC, which is still under construction, has good content validity, thanks to its extensive item collection. While most items of the scales are based on existing scales appropriate for other populations, the PACSLAC developers collected items that are characteristic of pain in elderly people with dementia. A preliminary checklist of pain behaviours was created based on interviews with professional long-term caregivers of older adults with severe communicative limitations due to dementia. In the second part of the study, nurses were asked to complete the checklist with reference to the pain experienced by a senior under their care. The current version is a long list, consisting of 60 items covering four sub-scales (facial expressions; activity/body movements; social/personality/mood; physiological/eating/sleeping/vocal), which were composed on the basis of item analysis. The underlying factor structure remains to be analysed. Examples of the items included in the scale are 'opening mouth', 'pacing', 'verbal aggression' and 'changes in sleep'. The items are scored if the behaviour is present. No scoring interpretation is currently available. The third part of the study focused on the preliminary validation of PACSLAC. High levels of internal consistency were found for the total scale (α = 0.82–0.92), although Cronbach's α values for the subscales were lower (.55–.73). The PACSLAC total score seemed to discriminate between painful, calm and distressing events. Correlations calculated between global intensity ratings and PACSLAC scores were moderate (r = .39–.54). Inter-correlations between the subscales suggest that although the checklist measures a unified construct, the subscales are sufficiently discriminatory [48].

Additional refinement and psychometric testing (test-retest, intra-rater reliability and factor analyses) of the PACSLAC is essential. This should include an assessment of its value in clinical situations and in larger samples. The checklist is long and covers a broad range of possible pain cues, included specifically for elderly people with limited communication abilities due to dementia. A major disadvantage is the fact that no patients participated directly in the studies undertaken to construct the scale. Instead, participating caregivers reported from memory on patients they had cared for [48]. It is questionable whether it is realistic to ask people to score a list of 60 items from memory. A final comment concerns the sample size involved in the study by Fuchs-Lacelle & Hadjistavropoulos that was used to construct the scale. Given the fact that the checklist contains many items, a sample size of 40 (recalled) patients seems inadequate.

The *Pain Assessment IN Advanced Dementia Scale (PAINAD) *by Warden *et al *(2003) was developed to assess pain in individuals with advanced dementia [49,50]. The scale can be described as a modification of the DS-DAT and was based on a review of the literature, available pain assessment tools (FLACC by Merkel et al., 1997 [51] and DS-DAT by Hurley et al., 1992 [30]) and consultation with expert clinicians. Testing was done in a residential setting (dementia care ward) involving 19 severely impaired patients. The current version consists of five items with three response modalities scored from 0 to 2 (with a range for the total scale of 0 to 10). Increasing levels reflect increasing degrees of pain. Examples of response modalities included in the 'facial expression' item are 0 = smiling; 1 = sad, frightened, frowning; 3 = facial grimacing [49,50]. Internal consistency was moderate and lower than desired (α < .70). Given the fact that the scale contains only a limited number of items (N = 5), the IC score is remarkably low. High levels of inter-rater reliability were found (Pearson r = .82–.97). The scale showed evidence of construct validity. The tool correlated well with the DS-DAT, VAS for discomfort and a VAS for pain. Pain scores were found to be lower during pleasant than during aversive activities and scores differed before and after pain modification. Factor analysis showed that there was one underlying construct, and item-total correlations were also investigated [49,50]. However, sample sizes used in developing PAINAD were small (N = 19), which limits its findings. Furthermore, pain scores were often clustered around 0, reflecting absence of pain. Since this might be a worrying aspect, further research should test the scales in more standardized pain situations in order to develop an adequate pain scale. Notwithstanding its good preliminary psychometric quality, PAINAD needs to be further tested (including test-retest reliability) in a larger sample. A training session is needed before the PAINAD scale can be used, and a manual is provided. Before applying the scale, a 5-minute observation period is required. The authors claim the scale to be user-friendly.

*The Pain Assessment for the Dementing Elderly (PADE) *by Villanueva et al. (2003) was developed to measure pain in individuals with advanced dementia [52]. The scale was developed after a literature review, interviews with nursing staff and observations. Testing was conducted in a residential setting (long-term care facilities) involving a sample of elderly people (N = 65) with mostly severe dementia. The PADE consists of 24 items covering three individual parts, the first assessing facial expressions, the second activities of daily living and the third the overall caregiver's judgement of pain. Examples of the items included in the scale are 'restless', 'frowning' and 'time spent out of bed'. The items are rated using several different scoring methods. While some items are rated on a four-point Likert scale, others are multiple choice and some items are scored on a VAS. While some items are scored retrospectively, others are not. Scoring interpretation is absent and the scale seems complex because it includes different scoring methods, which might be confusing or difficult to interpret. Therefore its clinical utility needs to be determined at the bedside. Because the scale includes different scoring methods, it seems problematic to calculate cut-off scores for pain and determine sensitivity and specificity. Considering the comprehensiveness of the tool, the number of participants was small. Several psychometric aspects have been investigated in a two-part study. Inter-rater reliability was found to be adequate (intra-class reliability .54–.96) while test-retest reliability was acceptable for most parts but low (intra-class reliability .34) for part 2 of the scale. Scores for the homogeneity of the scale were good for most parts of the scale, except for part 3. Results show that the second part of the scale is the most problematic part in terms of reliability. When correlated with a scale to measure agitation, the scale demonstrated a relation as hypothesized. The scale also provided evidence of construct validity by differentiating between pain and no-pain groups, but the construct validity of the scale needs to be further investigated.

Although it seems a long list, authors stated that, with practice, PADE requires 5–10 minutes to complete [52]. Given the scoring complexity, however, this is probably an underestimation.

*Rating Pain in Dementia (RaPID) *by Sign and Orrell (2003) was developed to rate pain in elderly people with dementia. It was developed from expert advice (N = 38 experts) and reviewed research literature [53]. It consists of 18 items covering four dimensions (behavioural, emotional, autonomic and postural). No specific information is provided about the origin of the items. The items are clustered and sometimes broadly defined. Examples of the items include 'tense body language', 'tearfulness', 'sweating' and 'general increase in muscle tone'. Items can be scored on a four-point scale (0 absent to 3 severe). The total score of the scale ranges from 0 to 54. Testing was done in a hospital setting (psychiatric and medical care units) involving 48 demented patients.

Observers score each item based on complaints, symptoms and signs occurring during one week, prior to using clinical judgements from a range of information such as clinical notes and interviews with staff, patient, and carer. To establish concurrent validity, RaPID scores were compared with the McGill Pain Questionnaire and a VAS. Findings showed that the instruments correlated highly with each other. In addition, good internal consistency of the total scale (α = .79) and good inter-rater reliability were found (mean .97). Similar high scores were found for test-retest reliability (ranging from .84 to .98) [53]. Closer examination of the data collection process on which this scale was based also yielded many pain scores clustered around 0. Investigation of the psychometric quality has so far been superficial, so this quality needs to be further investigated in larger samples. Based on these preliminary findings, further development of this scale seems warranted.

The *Abbey Pain Scale *by Abbey et al. is a brief assessment scale for people with end-stage dementia [54]. The scale is based on the pain scales described by Hurley et al. [30] and Simons & Malabar [39] and modified by geriatric and pain experts by means of a Delphi study [54]. The scale consists of 6 items (e.g. physiological changes, physical changes) with four response modalities scored from 0 (absent) to 3 (severe), with a range for the total scale of 0–18. The scale was tested in residential care facilities. After completing the observations and adding up the scores, the interpretation is as follows: '>3' mild pain, '8–13' moderate pain, '>14' severe pain. These cut-off scores are based on cross-tabulation of the Abbey pain scores against the holistic pain impression of the participating nurses (named holistic measure). To establish construct validity, scores were compared with nurses' overall pain impression. Findings showed that these scores correlated moderately (.59) with each other. Furthermore, pain scores were found to be lower after the intervention. Adequate levels of internal consistency were found for the total scale (α = 0.74–0.81), but low inter-rater reliability scores (scoring N = 18 patients) were found and test-retest reliability was not reported. Although several psychometric aspects have thus been tested, the current version of the pain instrument still lacks reliability and validity.

*The Non-Communicative Patient's Pain Assessment Instrument (NOPPAIN) *by Snow et al. (2004) consists of four sections and combines information about pain behaviour (words, noises, facial expression, bracing and restlessness), care conditions and a Likert scale of pain intensity [55]. Information about the origin of the items has not been clearly provided. After an initial feasibility study, the preliminary version of the NOPPAIN was tested in a small sample of 21 nursing assistants (NA). The researchers used a video gold standard method to portray a patient's painful situation during care. The recently published study [55] focuses on the validity of NA pain intensity scores compared to the video gold standard. The authors reported excellent agreement (kappa .87), providing preliminary evidence of construct validity. The scale might present a useful contribution but has not been extensively tested for validity or reliability. According to the authors, the scale is easy to administer (requiring very little training) and brief, and combines text and pictures to make it easier to understand. By focusing on nursing assistants, the developers underline the importance of pain assessment during daily care by key figures in nursing home care. However, it is questionable whether nursing assistants are capable of assessing a complex problem like pain during daily care situations. Evidence of validity and generalizability might be limited because developers created an artificial situation using a video approach (acting out a painful situation).

Davies et al. (2004a; 2004b) recently developed the *pain assessment scale for use with cognitively impaired adults *[56,57]. The scale was developed based on literature analysis and expert focus group discussions. While most assessment strategies have focused on one aspect of pain (e.g. pain intensity), these researchers tried to incorporate several pain aspects into one multidimensional tool which focuses on the assessment of existing painful conditions, physiological measures of pain, self-report, facial expression, usual pain behaviour and changes from usual behaviour. As a result, the current version, covering 11 sections, is very comprehensive. The sections/items are rated using different scoring methods.

The clinical utility of the scale has been pilot-tested in a small sample (N = 27 cognitively impaired elderly patients of a dementia care unit and a psycho-geriatric unit) by implementing the scale in practice over a three-month period. The tool was often not fully completed by respondents and was reported to be complex and time-consuming. There was a strong tendency to skip the section that relates to physiological assessment strategies, like blood pressure [57]. Before further determining the utility of the scale, it needs to be refined and tested for validity and reliability.

Table 3 presents the scores for individual criteria, as well as overall quality judgements, which reveal that the quality of the scales we have reviewed is generally moderate. Only four of the scales scored 11 points on our quality judgement which has a scoring range from 0 to 20, viz. DOLOPLUS2, ECPA, PAINAD, PACSLAC. It must be taken into account, however, that most of the scales are still under construction, especially with regard to criterion and construct validity. Future publications will probably highlight more research and psychometric findings.

## Discussion and Conclusion

The purpose of the present study was to review behavioural pain assessment tools available to assess pain in elderly people with severe dementia, and to evaluate the psychometric quality of these tools.

This systematic review revealed that at least 12 behavioural pain assessment tools currently exist. We conclude from the results of our review that at present, none of these assessment scales is convincingly the most appropriate, and therefore preferable, scale for assessing pain in elderly people with dementia. Our findings (based on quality judgement criteria relating to validity, reliability and homogeneity) demonstrate that PAINAD, PACSLAC, DOLOPLUS2 and ECPA show the best psychometric qualities. It should be stressed, however, that none of these tools scored more than 12 points out of a maximum quality score of 20, so their overall psychometric quality can be regarded as moderate. The tools therefore still await confirmation of various aspects of their psychometric properties.

Our review of the studies on behavioural assessment scales identified several general issues and weaknesses that need to be addressed, including methodological issues and practical limitations. First, to achieve the required validity, most instruments were correlated with a VAS or alternative intensity scale filled in by a proxy (mostly nurses). In the absence of self-reports, the interpretation of pain by a significant other has been frequently discussed, and the legitimacy of this approach (using a self-report scale by proxy as a gold standard or acceptable silver standard) is questionable. The assumption that caregivers' pain impression can be quantified by tools like VAS could only be legitimated if nurses' perceptions are comparable to patients' own perception of pain. Differences in pain rating between nurses and patients have been identified as an issue affecting pain measurement and management in elderly people [58]. If nurses' pain impression was a valid and reliable measure, a more complex behavioural scale to assess pain would become redundant. Instead of using a proxy report approach, an option could be to use a selected group of elderly people with opportunities to self-report their pain as an alternative strategy to further validate behavioural scales. Nonetheless, the scoring of observational tools also depends largely on pain perception by proxies. Although it remains a methodological pitfall, proxy reporting is often a valuable option in this population.

There are also some methodological concerns about sample sizes and the indicators collected to construct pain scales. We must be aware of the fact that the pain indicators collected for this purpose may be influenced by the type of pain focused on in collecting the items and the setting in which indicators are collected. Given the fact that some scales contain a large number of items, many of the studies used small samples of participants or a limited number of pain situations. In addition, articles do not always provide information about the frequency of endorsement of certain items in the population examined. Not providing information about the importance of items at rest and during a painful situation can affect results. Furthermore the current scales are heterogeneous in terms of items used to assess pain. The overlapping items of the scales might be the most common and important ones, while unusual items might be more characteristic of the target group but less useful for a general pain scale for elderly people with dementia. In other words, item responsiveness to pain adds to further item reduction and refinement of a scale.

In view of the limited qualities of the scales, including PAINAD, PACSLAC, DOLOPLUS2 and ECPA, further research is essential for additional refinement and development. It may therefore be questioned if recommendations can at this stage be made for the implementation of one of these tools in clinical practice. In answering this question, two further criteria could be added.

The first criterion concerns the ability of items in the scale to detect subtle changes in behaviour. These specific items add important information and help nurses to create a certain pain image of the non-verbal patient. Therefore, we would expect that indicators which focus on subtle behaviours should be adequately covered by the items in the pain scale. While PAINAD, DOLOPLUS2 and ECPA tend to focus on main indicators like facial expression, PACSLAC is the only scale that primarily focuses on these subtle changes in behaviour. Notably, PACSLAC is one of the few instruments in which the item collection is based on pain items specifically geared towards elderly persons with dementia, instead of items adjusted from existing scales developed for use in different patient groups (like paediatrics). In view of the special needs of the heterogeneous group of elderly people with dementia, this is a more suitable procedure to use in creating an item bank specifically for the target group.

The second criterion relates to the clinical utility of the scales. Ramelet et al. [59] stated that clinical utility and feasibility are of paramount importance for the acceptability of a measure in clinical practice. Authors often claim good clinical utility even though these aspects have not been properly evaluated. Scoring method, number of items and scoring interpretation are factors that must be considered in valuing an instrument's utility. Available evidence of clinical utility is scarce and criteria for scoring and interpreting scores are often not available. Furthermore, most studies lack information on sensitivity and specificity, and without this information, a scale is useless for clinical practice. This major limitation must receive more attention, which means that further testing in clinical practice is needed. Having an instrument tested in nursing home practice by nurses adds to the body of knowledge about its real utility value. It must be concluded that none of the behavioural pain assessment scales have been extensively tested in a variety of care settings under different pain conditions by various caregivers, which means that so far they cannot be said to have good clinical utility. None of the scales has thus proved practicable enough to be used in clinical situations like nursing practice on a daily basis. Of the four highest scoring scales, DOLOPLUS2 has been most comprehensively tested.

After adding these criteria to the psychometric properties, we conclude that PACSLAC and DOLOPLUS2 are the most appropriate scales currently available.

### Limitations of the study

Before recommendations for further research can be formulated, there are some limitations of the present systematic review that need to be addressed. To begin with, it must be noted that the studies reviewed above show considerable heterogeneity in terms of design (retrospective vs. prospective), method (pain in vivo vs. observational methods), research population (different types of dementia, different levels of impairment, different settings) and conceptualisation of pain, making their results hard to compare. Aspects that make the studies difficult to compare include differences in format/structure and scoring method. DOLOPLUS2, PADE and PACSLAC, for example, are extremely different in these respects. Although we used a set of criteria to arrive at an overall judgement to make our review more objective and systematic, quality judgement scores should always be interpreted with caution, because the use of some criteria inevitably involves a subjective element derived from the reviewer's expertise. Each criterion used to evaluate the quality of the scales was given an equal weight of 1 (except for construct validity, which was given a weight of 2, based on perceived importance). It is important to realize that this weighting approach inevitably has consequences for the quality judgement scores.

### Recommendations and further research

The findings of this review have important implications for future research and for everyday practice. Pain assessment is recognized as a significant area for future research and for the improvement of nursing care [60]. Pain assessment fits into a broader perspective of evaluating elderly people's daily functioning and quality of life, which is the core business for nurses. Assessment and reassessment lead to accurate and regular documentation of pain scores, which is extremely important in the evaluation and continuity of daily care.

Although huge progress has been made over the last decade, and studies of pain assessment among cognitively impaired elderly people have yielded promising results, studies have so far been limited. Assessment in the severely demented elderly remains difficult, and diagnosing pain continues to be a daily challenge to nurses. Although using a pain assessment scale is an important resource in detecting pain, it is often an element of a more comprehensive approach that also uses other resources, like physical examination and information from close relatives. These explorations of various resources can add information to solve the pain problem and therefore remain necessary.

In recent years, there has been growing interest in pain among cognitively impaired elderly people, which is illustrated by the fact that more than half of the 12 scales included in this review were published after 2002. Evidently, the number of newly developed scales has grown very rapidly. In view of this proliferation of behavioural tools and the promising quality of some of the scales reviewed here, we recommend improving these scales on the basis of further testing of their validity, reliability and clinical utility. It is the researchers' as well as the funding agencies' and journals' responsibility to prevent excessive growth of newly developed tools. Thus, further psychometric evaluation of existing scales should be given priority over developing new scales for future use. Valid, practical and reliable scales can add to the body of knowledge about pain and help to improve pain treatment in this important and growing population.

Scherder and colleagues [21,61,62] concluded that the type of dementia seemed to influence pain reports. This might actually imply a validity issue regarding the use of specific behavioural indicators across different stages and types of dementia. Knowing that the type and stage of dementia does matter in relation to pain assessment, further research should determine the utility, validity and reliability of pain assessment using a pain scale that takes the type of dementia into account. Furthermore, the results of the various studies show that there has been little research addressing the effect of cultural background on pain. Since none of the reviewed pain assessment scales seriously considers this variable, this is another aspect that should be included in future research.

A final recommendation concerns the Behavioural and Psychological Symptoms of Dementia (BPSDs) in relation to pain. BPSDs can confound pain assessment. Until now, little is known about the interaction between pain symptoms and these behavioural problems. Therefore, the relation between pain and BPSDs needs to be explored. Further research will be needed to determine its sensitivity in relation to these other concepts, as well as the way pain affects these symptoms and how these symptoms affect pain expression.

## Competing interests

The author(s) declare that they have no competing interests.

## Authors' contributions

All authors critically reviewed the manuscript, read and approved the final manuscript.

## Pre-publication history

The pre-publication history for this paper can be accessed here:


